# Quality of life and mental health status of glaucoma patients

**DOI:** 10.3389/fmed.2024.1402604

**Published:** 2024-06-03

**Authors:** Vanja Kopilaš, Mirko Kopilaš

**Affiliations:** ^1^Faculty of Croatian Studies, University of Zagreb, Zagreb, Croatia; ^2^Private Ophthalmology Clinic, Dubrovnik, Croatia

**Keywords:** glaucoma, quality of life, mental health, depression, anxiety

## Abstract

**Introduction:**

Glaucoma, a leading cause of irreversible blindness worldwide, poses significant challenges to patients’ quality of life (QOL) and mental well-being.

**Methods:**

This study aimed to investigate the complex interplay between clinical, demographic, and psychological factors and their impact on QOL among patients diagnosed with glaucoma. A cohort of 201 glaucoma patients, with a mean age of 70 years, participated in the study.

**Results:**

Descriptive analyses revealed that participants reported living with a glaucoma diagnosis for an average of 13.38 years, highlighting the chronic nature of the disease in the cohort. Comorbidity was shown to be in close relationship with QOL, where with additional health problems have lower QOL scores (M = 34.86, SD = 18.25), as well as higher levels of anxiety (M = 10.64, SD = 5.38) and depression (M = 13.42, SD = 7.37). Correlation analyses further unveiled robust associations between clinical characteristics and psychological outcomes, with lower visual acuity strongly correlated with reduced QOL (rR = −0.74, pR < 0.001; rL = −0.78, pL < 0.001) and higher levels of anxiety and depression. Additionally, longer duration of glaucoma diagnosis was moderately associated with poorer QOL (r = 0.56, *p* < 0.001) and increased psychological distress, highlighting the cumulative burden of living with the disease over time. Mediation analyses indicated that duration of diagnosis partially mediated the relationship between depression and QOL, as well as anxiety and QOL, suggesting that the prolonged experience of living with glaucoma may exacerbate the impact of psychological distress on QOL.

**Discussion:**

These findings underscore the importance of holistic patient care approaches that address both the physical and psychological aspects of glaucoma to improve patient outcomes and enhance overall well-being.

## 1 Introduction

Vision is integral to nearly every aspect of daily life, facilitating essential activities such as navigation, communication, and personal independence (1, 2). Thus, any impairment to vision can have profound consequences on an individual’s well-being and quality of life (QOL). Glaucoma, a group of progressive optic neuropathies characterized by damage to the optic nerve and visual field loss, represents one such condition that poses significant challenges to affected individuals (3, 4). Despite its asymptomatic nature in the early stages, glaucoma can lead to irreversible vision loss if left untreated, highlighting the critical importance of early detection and intervention in preserving visual function (5). The impact of glaucoma extends beyond its ocular manifestations, permeating various aspects of everyday living (6). As the disease progresses, individuals may experience limitations in performing routine activities, such as driving, reading, and participating in social events (7). These changes can result in feelings of frustration, dependence, and diminished QOL (8–10). Furthermore, the psychosocial consequences of glaucoma are profound, with vision loss often precipitating emotional distress, anxiety, and depression (7, 11, 12). Symptoms of depression and anxiety are often reported in glaucoma patients, and some research even consider them as potential risk factors for glaucoma development (13–15). Patients diagnosed with open-angle glaucoma frequently encounter challenges in identifying facial expressions and have an increased susceptibility to feelings of depression and anxiety, commonly linked to the deterioration of their vision (16, 17). Nevertheless, the temporal pole, a component of the limbic system in conjunction with the amygdala that is known to be involved in several functions such as such as emotion and behavior, is also involved in the processes of facial recognition and memory storage (18, 19). Understanding the complex interplay between clinical, demographic, and psychological factors and their impact on QOL among glaucoma patients is essential for informing targeted interventions aimed at improving patient outcomes. Previous research has highlighted the significant associations between comorbidity, disease duration, psychological distress, and QOL in this population (8–10, 14, 20–22). However, further investigation is warranted to elucidate the underlying mechanisms driving these relationships and to identify potential modifiable factors that can be targeted in clinical practice. Theoretical framework is provided with biopsychosocial model that is supporting the notion that certain medical conditions can only be understood through the examination of the interaction between physiological, psychological, and sociocultural factors (23, 24). Therefore, the present study aims to comprehensively examine the multifaceted relationship between clinical, demographic, and psychological factors and their impact on QOL among patients diagnosed with glaucoma. Utilizing a different statistical methods including descriptive analyses, correlation analyses, independent sample *t*-tests, and mediation analyses, we seek to address the following objectives:

- Investigate the associations between comorbidity, disease duration, and psychological distress (anxiety and depression) with QOL among glaucoma patients.
- Examine the mediating role of disease duration in the relationship between psychological distress and QOL in glaucoma patients.

By addressing these objectives, our aim is to contribute to a deeper understanding of the determinants of QOL in glaucoma patients and to inform targeted interventions aimed at improving patient outcomes and enhancing overall well-being. Additionally, by elucidating the complex interplay of factors influencing QOL, this study seeks to advance our understanding of the biopsychosocial dimensions of glaucoma and to underscore the importance of holistic approaches to patient care.

## 2 Materials and methods

### 2.1 Participants

Two hundred and one participants enrolled in this cross-sectional study were individuals undergoing routine ophthalmological examinations, all of whom had received a diagnosis of primary open-angle glaucoma from their attending ophthalmologist. Ethical approval for this study was obtained by the Ethics Committee at the University of Zagreb Faculty of Croatian Studies (reference number: 053-01/23-2/0001), ensuring that the research complied with all relevant ethical standards and guidelines. These participants were recruited during their visits to the ophthalmology clinic and were invited to participate in the research study. Prior to their involvement, each participant signed informed consent, indicating their understanding of the study’s purpose, procedures, and their voluntary participation.

Upon obtaining informed consent, participants were guided through the study protocol by trained research personnel. The procedure involved the completion of a comprehensive battery of questionnaires, designed to assess various psychosocial and demographic factors pertinent to the experience of glaucoma.

### 2.2 Measures

The battery of questionnaires administered to participants included:

Glaucoma Quality of Life Questionnaire (GQL-15) (25): The GQL-15 is a validated instrument tailored to assess the impact of glaucoma on various aspects of an individual’s QOL, covering domains such as vision-related function, mobility, and emotional well-being. The GLQ-15 consists of 15 items. Each question has scores ranging from 0 to 5, where 0 is difficulty to perform the task due to non-visual problems, 1 is no difficulty, and 5 is severe difficulty. The highest score is 75 and the lowest is 15, where higher scores indicate more difficulties with vision-related activities and are associated with lower quality of life as well. The Cronbach alpha for the current study indicates good reliability of the GQL-15 (α = 0.986).

Generalized Anxiety Disorder-7 Scale (GAD-7) (26): The GAD-7 is a widely used self-report measure consisting of 7 items designed to assess the severity of anxiety symptoms experienced by individuals over the past 2 weeks, providing valuable insights into the prevalence and intensity of anxiety. Each item has scores ranging from 0 to 3, where 0 is not at all, 1 is several days, and 3 is nearly every day. The GAD-7 total score ranges from 0 to 21. The Cronbach alpha for the current study indicates satisfactory reliability of the GAD-7 (α = 0.95).

Patient Health Questionnaire-9 (PHQ-9) (27): The PHQ-9 is a reliable and validated tool for evaluating the severity of depressive symptoms. It consists of 9 items exploring the frequency and intensity of depressive symptoms experienced by participants. Each item explores presentation of certain experiences over the course of the last 2 weeks with scores ranging from 0 to 3, where 0 is not at all, 1 is several days, and 3 is nearly every day. The PHQ-9 total score ranges from 0 to 27. The Cronbach alpha for the current study indicates satisfactory reliability of the PHQ-9 (α = 0.968).

Sociodemographic Questionnaire: A structured questionnaire was administered to collect sociodemographic information from participants, including age, gender, educational background, employment status, medical information (duration of diagnosis, visual acuity, comorbidities) and other relevant demographic variables. This information allows for the characterization of the study sample and facilitates the exploration of potential associations between sociodemographic factors and the psychosocial variables under investigation.

The administration of these questionnaires was conducted in a standardized manner, ensuring consistency and reliability across participants. Participants were encouraged to provide accurate and honest responses to each item, with the assurance of confidentiality and anonymity maintained throughout the data collection process.

### 2.3 Data analysis

Data was analyzed with IBM SPSS Statistics program (version 26). Before conducting statistical analysis, the normality of the continuous variables was tested. The results of the Shapiro–Wilk test indicated deviations from the normality for all continuous variables. However, it should be noted that all the distributions prove to be symmetrical, except the distribution of quality of life. Descriptive analyses were conducted to compute means, and standard deviation of continuous variables, as well as the median and interquartile range for the variable of quality of life. Additionally, we examined proportions of categorical variables.

In order to examine the differences in observed variables (anxiety, depression, and quality of life) regarding gender and comorbidity, an independent sample *t*-test were performed.

To determine the associations between glaucoma related quality of life, measures of psychological distress (anxiety and depression), and certain aspects of participants’ health, the values of the Pearson correlation coefficient were observed. Significance was set at *p* < 0.05.

Mediation analyses were performed to assess whether years of living with glaucoma were significant mediator of the relationship between depression and quality of life, as well as anxiety and quality of life. PROCESS module in SPSS [version 4.0., Model 4, Hayes (28)] was used to conduct the above-mentioned analysis. Significance of the indirect effect was tested with bootstrap method, where confidence interval (CI) was set at 95% and based on 5,000 bootstrap samples. The indirect effect is considered statistically significant if the 95% CI does not contain a value of 0.

## 3 Results

### 3.1 Descriptive statistics

This study included 201 patients with glaucoma, of which 125 (62.2%), were women, and 76 (37.8%) were men. Participants were between the ages of 42 and 94, with the average age of 70 years (M = 70.4; SD = 11.52). Most of them marked high school as the highest level of education (42.8%), are retired (64.2%), married (56.7%) and have children (82.6%). On average, our participants live more than 13 years with a diagnosis of glaucoma (M = 13.38; SD = 7.73). Regarding visual acuity, most of them have normal sight on one of the eyes based on the International Classification of Diseases 11 (ICD-11) classification for distance vision impairment (29). Sample characteristics are presented in Table 1.

**Table 1:** Sociodemographic information of the participants (*N* = 201).

		*N* (%)
Gender	Female	125 (62.2)
	Male	76 (37.8)
Education	Elementary school	47 (23.4)
	High school	86 (42.8)
	University	68 (33.8)
Work status	Full-time work	55 (27.4)
	Unemployed	17 (8.5)
	Retired	129 (64.2)
Relationship status	Married	114 (56.7)
	Unmarried	24 (11.9)
	Widowed	53 (26.4)
	Divorced	10 (5.0)
Children	Yes	166 (82.6)
	No	35 (17.4)
Comorbidities	Yes	88 (43.8)
	No	113 (56.2)
Visual acuity- R	Blindness	14 (7.0)
	Severe	5 (2.5)
	Moderate	20 (9.95)
	Mild	17 (8.45)
	Normal	145 (72.1)
Visual acuity- L	Blindness	14 (7.0)
	Severe	7 (3.5)
	Moderate	19 (9.45)
	Mild	20 (9.95)
	Normal	141 (70.1)

	M (SD)
Age	70.4 (11.5)
Duration of diagnosis	13.38 (7.73)

The mean total score on the GQL-15 was 34.86 (SD = 18.25) indicating somewhat good quality of life of our glaucoma patients. Considering that the quality of life has a positively skewed distribution, it is necessary to consider the values of the median, which is 28 (interquartile range- iqr = 30), and deviates from the previously mentioned mean values. The mean score for depression on PHQ-9 was 13.42 (SD = 7.37), while the mean GAD-7 score was 10.64 (SD = 5.38). Using a cut-off score of ≥ 8 (30, 31), more than two thirds of the sample (68.3%) would be categorized as depressed, and approximately 63% (63.8%) would be considered anxious (30, 31).

### 3.2 Differences in the observed variables

The results of *t*-test (Table 2) indicated a statistically significant difference in quality of life, depression and anxiety regarding comorbidity, but not gender. Glaucoma patients with additional health problems reported greater difficulties in performing daily activities (GQL-15), and higher levels of anxiety and depression. Due to the positively skewed distribution of the quality of life, a Mann Whitney U test was performed (U = 7,143, z = 5.31, *p* < 0.001) which confirmed a significant difference in quality of life with respect to the comorbidity. In addition, measures of psychological distress (anxiety and depression) and quality of life differed significantly based on comorbidity, even after controlling for age. There were no statistically significant gender differences in observed variables, i.e., the GQL-15 scores, PHQ-9 scores and GAD-7 scores were similar for both groups. Additionally, observed variables did not differ significantly in regard to gender, after controlling for the age of participants.

**Table 2:** Differences in observed variables regarding gender and comorbidity.

Dependent variable	Group		*M* (SD)	*t* (df)	*p*-value	95% CI	
GQL-15	Gender	Female	33.18 (17.35)	−1.686 (199)	0.093	[−9.667, 0.756]	
		Male	37.63 (19.45)				
	Comorbidity	Yes	40.77 (15.95)	−4.219 (199)	**<0.001**	[−15.431, −5.601]	
		No	30.26 (18.67)				
PHQ-9	Gender	Female	13.21 (7.38)	−0.517 (199)	0.606	[−2.672, 1.561]	
		Male	13.76 (7.38)				
	Comorbidity	Yes	15.60 (6.43)	−3.835 (199)	**<0.001**	[−5.883, −1.888]	
		No	11.72 (7.62)				
GAD-7	Gender	Female	10.53 (5.4)	−0.367 (199)	0.714	[−1.836, 1.260]	
		Male	10.82 (5.39)				
	Comorbidity	Yes	12.26 (4.69)	−3.907 (199)	**<0.001**	[−4.348, −1.431]	
		No	9.37 (5.56)				

Significant values bolded. M, mean; SD, standard deviation; t, t-statistic; df, degrees of freedom; 95% CI, 95% confidence interval; GQL-15, Glaucoma Quality of Life Questionnaire; PHQ-9, Patient Health Questionnaire-9; GAD-7, Generalized Anxiety Disorder-7 Scale.

### 3.3 Correlation analysis

To determine the association between certain aspects of participants’ health (visual acuity, duration of diagnosis), and quality of life, anxiety and depression, Pearson’s correlation analysis was conducted. Quality of life was significantly negatively associated with observed clinical characteristics. Visual acuity in both eyes was significantly negatively associated with GQL-15 (Pearson’s correlation coefficient of the right eye- *r_R_* = −0.74, corresponding *p*-value of the right eye- *p_R_* < 0.001; Pearson’s correlation coefficient of the left eye *r_L_* = −0.78, corresponding *p*-value of the left eye- *p_L_* < 0.001), indicating that glaucoma patients with lower visual acuity tend to experience greater difficulties in performing daily activities. Furthermore, visual acuity exhibited significant negative correlation with depression (*r_R_* = −0.59, *p_R_* < 0.001; *r_L_* = −0.75, *p_L_* < 0.001) and anxiety (*r_R_* = −0.6, *p_R_* < 0.001; *r_L_* = −0.71, *p_L_* < 0.001). Duration of glaucoma diagnosis was moderately and positively associated with GQL-15 (*r* = 0.56, *p* < 0.001), PHQ-9 (*r* = 0.5, *p* < 0.001), and GAD-7 (*r* = 0.48, *p* < 0.001), implying that longer coexistence with glaucoma is linked to greater perception of difficulties in everyday functioning and higher levels of depression and anxiety. Additionally, decrease in performance-related quality of life (GQL-15) was significantly positively correlated with depression (*r* = 0.83, *p* < 0.001) and anxiety (*r* = 0.8, *p* < 0.001).

### 3.4 Mediation analysis

**Figure 1:**
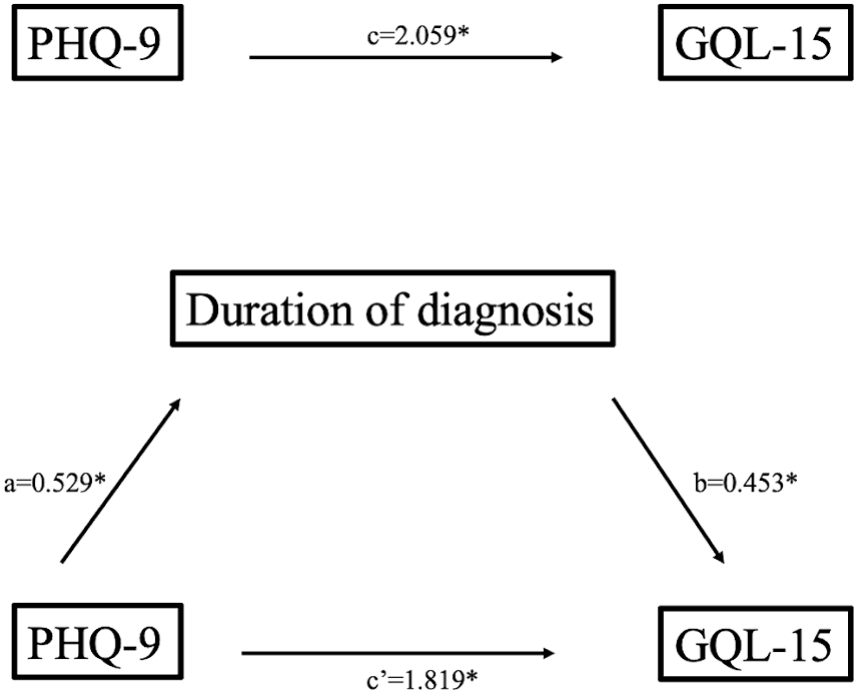
Mediation model of depression on quality of life through duration of diagnosis (unstandardized coefficients); *p < 0.001.

To assess the possible mediating role of duration of diagnosis in the relationship between anxiety and quality of life, as well as depression and quality of life, simple mediation analyses were performed. Regarding the first mediation model (Figure 1), the path coefficients from depression to duration of diagnosis (b = 0.53, *p* < 0.001; 95% CI [0.402, 0.655]), and from depression to quality of life (path c) were significant (b = 2.06, *p* < 0.001, 95% CI [1.866, 2.251]). As shown in Table 3, duration of the diagnosis made a significant contribution to quality of life (b = 0.45, *p* < 0.001). Considering the bootstrap 95% confidence interval, the significant indirect effect from depression to quality of life through duration of diagnosis was confirmed (b = 0.24, 95% CI [0.112, 0.401]). Finally, the direct effect of depression on quality of life remained significant, but the effect value was somewhat lower (b = 1.82, *p* < 0.001).

**Table 3:** Path estimates of mediation models.

Outcome	Predictors	B	SE	*β*	95% CI	*R* ^2^	*F*	*p*-value
GQL-15	PHQ-9	1.82	0.11	0.73	[1.61, 2.03]	0.718	251.94	**<0.001**
	DD	0.45	0.1	0.19	[0.25, 0.65]			
	PHQ-9 x DD	0.24	0.07	0.097	[0.11, 0.401]			
GQL-15	GAD-7	2.35	0.15	0.69	[2.05, 2.65]	0.685	215.18	**<0.001**
	DD	0.54	0.11	0.23	[0.33, 0.75]			
	GAD-7 x DD	0.37	0.11	0.11	[0.19, 0.59]			

Significant values bolded; B, unstandardized coefficient; SE, Standard Error; β, standardized (beta) coefficient; 95% CI, 95% confidence interval; R^2^, coefficient of determination; F, F- statistic; DD, duration of diagnosis; GQL-15, Glaucoma Quality of Life Questionnaire; PHQ-9, Patient Health Questionnaire-9; GAD-7, Generalized Anxiety Disorder-7 Scale.

**Figure 2:**
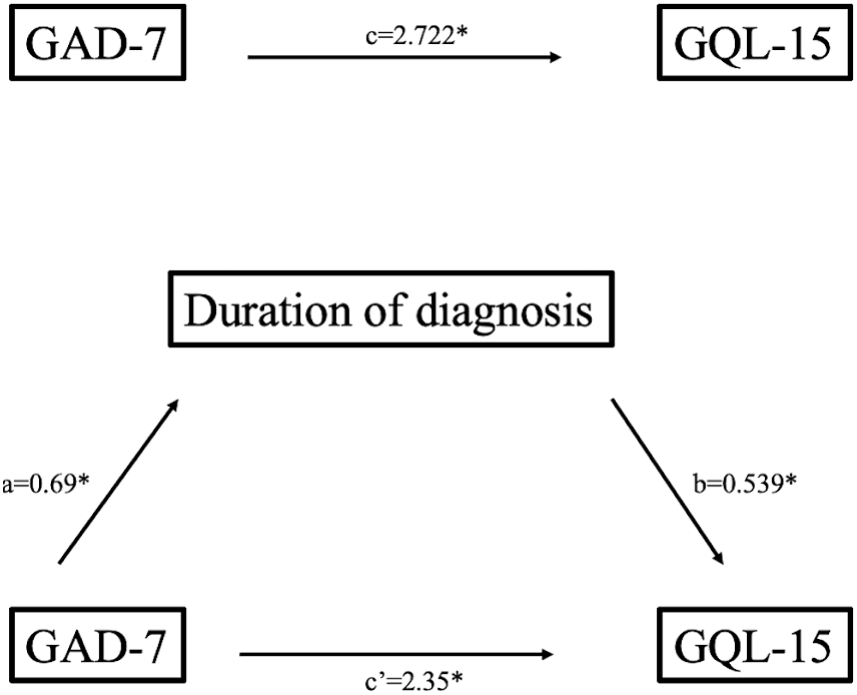
Mediation model of anxiety on quality of life through duration of diagnosis (unstandardized coefficients); *p < 0.001.

Regarding the second mediation model (Table 3; Figure 2), anxiety made significant contribution to duration of the diagnosis (b = 0.69, *p* < 0.001, 95% CI [0.515, 0.866]), and to quality of life (path c, b = 2.72, *p* < 0.001, 95% CI [2.44, 3.01]). Duration of diagnosis positively predicted vision related quality of life (b = 0.54, *p* < 0.001). Moreover, the indirect effect of anxiety on quality of life through duration of diagnosis (b = 0.37, *p* < 0.001) was also significant. After accounting for duration of diagnosis as mediator, the direct effect of anxiety on quality of life remained significant (b = 2.35, *p* < 0.001), indicating that years living with diagnosis partially mediates the relationship between level of anxiety and vision related quality of life in glaucoma patients.

## 4 Discussion

Our study employed an analytical approach to investigate the complex interplay between clinical, demographic, and psychological factors and their impact on the QOL among patients diagnosed with glaucoma. Series of analyses was conducted, including descriptive statistics, correlation analyses, independent sample *t*-tests, and mediation analyses with specific aim of exploring the underlying mechanisms behind the observed relationships.

Descriptive analyses revealed important demographic characteristics of our sample, comprising 201 glaucoma patients, with 62.2% being women and 37.8% men. The average age of participants was 70 years, with the majority having completed high school education (42.8%), being retired (64.2%), married (56.7%), and having children (82.6%). Furthermore, the participants reported living with a glaucoma diagnosis for an average of 13.38 years, highlighting the chronic nature of the disease in our cohort.

Our analysis of clinical measures revealed significant associations between comorbidity and QOL among glaucoma patients. Those with additional health problems reported significantly lower QOL scores (M = 34.86, SD = 18.25), as well as higher levels of anxiety (M = 10.64, SD = 5.38) and depression (M = 13.42, SD = 7.37). Notably, more than two-thirds of the sample would be categorized as depressed, and approximately 63% would be considered anxious, indicating a high prevalence of mental distress in this population.

While our study did not find statistically significant gender differences in QOL, anxiety, or depression among glaucoma patients, both male and female participants exhibited similar scores on these measures. This suggests that gender may not be a significant predictor of psychological well-being in this population. Contrary, previous research found that women with glaucoma were more likely to experience depression and stress (22). However, research is not quite clear about the role of gender suggesting that this area has yet to be explored. Our findings are in line with other research showing no significant gender difference among glaucoma patients (32).

Correlation analyses unveiled robust associations between clinical characteristics and psychological outcomes. Lower visual acuity was strongly correlated with reduced QOL (*r_R_* = −0.74, *p_R_* < 0.001; *r_L_* = −0.78, *p_L_* < 0.001) and higher levels of anxiety (*r_R_* = −0.6, *p_R_* < 0.001; *r_L_* = −0.71, *p_L_* < 0.001) and depression (*r_R_* = −0.59, *p_R_* < 0.001; *r_L_* = −0.75, *p_L_* < 0.001), emphasizing the profound impact of visual impairment on psychosocial functioning. This is in line with previous research confirming significant decrease in social functioning and mental health due to the lower visual acuity (33, 34). Additionally, longer duration of glaucoma diagnosis was moderately associated with poorer QOL (*r* = 0.56, *p* < 0.001) and increased psychological distress, highlighting the cumulative burden of living with the disease over time. These results are expected. Progression of glaucoma symptoms is expected to have an effect on the psychological well-being and everyday functioning (14, 35). Changes imposed by the lower vision quality can lead to the higher prevalence of anxious and depressive symptoms, and then consequently to the lower QOL.

Our mediation analyses provided further insights into the underlying mechanisms linking anxiety, depression, and QOL in glaucoma patients. Duration of diagnosis emerged as a significant mediator, partially mediating the relationship between anxiety and QOL, as well as depression and QOL. Specifically, the indirect effect of anxiety on QOL through duration of diagnosis was significant (b = 0.37, *p* < 0.001), suggesting that the prolonged experience of living with glaucoma may exacerbate the impact of anxiety on QOL. These findings confirm previous research showing existent triad of factors impacting QOL (14, 36, 37). As such, it becomes imperative for healthcare systems to prioritize interventions targeting these areas to enhance QOL for individuals living with glaucoma. By addressing the complex interactions between anxiety, depression, and duration of diagnosis, healthcare providers can implement more effective strategies to improve the well-being of glaucoma patients.

Our study has several practical significances. Based on the empirical evidence, new prevention and curation programs can be developed in order to improve relationships between clinical, demographic, and psychological factors in glaucoma patients. By identifying key determinants of QOL and psychological distress, our findings underscore the importance of holistic patient care in managing this complex condition. Future research employing longitudinal designs and objective assessments could provide further insights into the dynamic interplay between these factors, ultimately informing targeted interventions to improve the well-being of individuals living with glaucoma.

### 4.1 Limitations

There are certain limitations related to this study. Due to the cross-sectional design, our findings have to be carefully interpreted. In order to have more generable conclusions, it would be beneficial to run longitudinal study looking at how these variables may change over time, and whether that change is related to the interplay between observed variables, or some other factors. One of the potential limitations is definitely gender ratio of our sample, and that majority of participants were older. Future research should make sure that sample is fair representative of the population in terms of gender and age. Finally, differences between various glaucoma types were not observed. Rather, we were interested if one has diagnosis of glaucoma or not. In order to see if different glaucoma types differ in the relationship with observed variables, future studies should incorporate this in the study design.

## 5 Conclusion

Our study explored notable impacts of comorbidity on the quality of life (QOL) among glaucoma patients, accompanied by increased levels of anxiety and depression. Although gender disparities in QOL were not notable, strong associations were discovered between clinical factors such as visual acuity and duration of diagnosis with QOL and psychological distress. These findings emphasize the significance of comprehensive patient care and propose the necessity for targeted interventions to relieve the mental burden experienced by glaucoma patients. Future exploration employing longitudinal designs could provide further insights into effective management strategies for this intricate condition.

## Data availability statement

The raw data supporting the conclusions of this article will be made available by the authors, without undue reservation.

## Ethics statement

The studies involving humans were approved by Ethics Committee at the University of Zagreb Faculty of Croatian Studies. The studies were conducted in accordance with the local legislation and institutional requirements. The participants provided their written informed consent to participate in this study. Written informed consent was obtained from the individual(s) for the publication of any potentially identifiable images or data included in this article.

## Author contributions

VK: Conceptualization, Formal analysis, Methodology, Writing – original draft, Writing – review & editing. MK: Investigation, Methodology, Resources, Supervision, Writing – review & editing.
